# Noninvasive Method for Measuring Local Pulse Wave Velocity by Dual Pulse Wave Doppler: In Vitro and In Vivo Studies

**DOI:** 10.1371/journal.pone.0120482

**Published:** 2015-03-18

**Authors:** Zhen Wang, Yong Yang, Li-jun Yuan, Jie Liu, Yun-you Duan, Tie-sheng Cao

**Affiliations:** Department of Ultrasound Diagnostics, Tangdu Hospital, Fourth Military Medical University, Xi’an, China; Shanghai Institute of Hypertension, CHINA

## Abstract

**Objectives:**

To evaluate the validity and reproducibility of a noninvasive dual pulse wave Doppler (DPWD) method, which involves simultaneous recording of flow velocity of two independent sample volumes with a measurable distance, for measuring the local arterial pulse wave velocity (PWV) through in vitro and in vivo studies.

**Methods:**

The DPWD mode of Hitachi HI Vision Preirus ultrasound system with a 5–13MHz transducer was used. An in vitro model was designed to compare the PWV of a homogeneous rubber tubing with the local PWV of its middle part measured by DPWD method. In the in vivo study, local PWV of 45 hypertensive patients (25 male, 49.8±3.1 years) and 45 matched healthy subjects (25 male, 49.3±3.0 years) were investigated at the left common carotid artery (LCCA) by DPWD method.

**Results:**

In the in vitro study, the local PWV measured by DPWP method and the PWV of the homogeneous rubber tubing did not show statistical difference (5.16 ± 0.28 m/s vs 5.03 ± 0.15 m/s, p = 0.075). The coefficient of variation (CV) of the intra- and inter- measurements for local PWV were 3.46% and 4.96%, for the PWV of the homogeneous rubber tubing were 0.99% and 1.98%. In the in vivo study, a significantly higher local PWV of LCCA was found in the hypertensive patients as compared to that in healthy subjects (6.29±1.04m/s vs. 5.31±0.72m/s, P = 0.019). The CV of the intra- and inter- measurements in hypertensive patients were 2.22% and 3.94%, in healthy subjects were 2.07% and 4.14%.

**Conclusions:**

This study demonstrated the feasibility of the noninvasive DPWD method to determine the local PWV, which was accurate and reproducible not only in vitro but also in vivo studies. This noninvasive echocardiographic method may be illuminating to clinical use.

## Introduction

The arterial pulse wave velocity (PWV) is defined as the speed of pressure pulse wave generated by the contraction of the left ventricle propagating down the arterial tree. This velocity is proportional to the square root of the elastic modulus, representing the arterial stiffness, according to the Moens-Korteweg formula [1]. Carotid-femoral PWV is accepted as the ‘gold-standard’ measurement of arterial stiffness, which has proven its independent predictive value for cardiovascular events in the elderly, hypertensive, diabetics, and patients with chronic renal failure and coronary heart disease as well as general population [2–9]. Aortic PWV is now of utmost importance. As we know, the aortic PWV only provides the average PWV over a long segment composed of arteries with different mechanical characteristics and with a major source of inaccurate estimation due to the poor distance measurement on the body surface[10, 11]. It may cause inaccuracy when local arteries are differently affected by aging and some diseases, for example, in early stage atherosclerosis [12]. Therefore, great emphasis has been placed on local or regional PWV in recent years [13, 14], since it may also provide some diagnostic information of biomechanical properties for local artery wall, the mechanical characteristics of which vary along the arterial tree. And they are differently affected by aging and disease. The functional properties of the local large arteries can be analyzed by noninvasive techniques such as echo-tracking [15], ultrasound [16, 17], magnetic resonance imaging [18, 19], or invasive techniques. However, among the current noninvasive methods, none could be directly used to measure local PWV with accuracy and simplicity in clinical settings.

This study presents a noninvasive method for measuring the local arterial PWV by using a clinical existing dual pulse wave Doppler (DPWD) echocardiographic technology. This technology enables us simultaneously record blood flow velocity spectra at two different nearby sites with validation in our previous studies [20, 21]. We hypothesized that local arterial PWV could be obtained by distance measurement of the observed segment divided by the transit time of the pulse wave on the DPWD spectra. In this in vivo study, the carotid artery was chosen to investigate because local carotid stiffness may provide some prognostic information, since it is a frequent site of atheroma formation.

The objective of this study was to evaluate the feasibility and reproducibility of the DPWD method for measuring local PWV. This study addresses the following issues: (1) the validation of the accuracy and reproducibility of this method using an in vitro experiment; (2) its clinical applicability and reproducibility on human subjects by performing the in vivo study; (3) its capability to differentiate groups of healthy and hypertension middle-aged subjects by means of the local PWV of the left common carotid artery (LCCA).

## Methods

### Echography

A Hi Vision Preirus ultrasonography system (Hitachi) with a 5–13 MHz linear array transducer (EUP-L74M) was used in the in vitro and in vivo studies. The PW/PW function of the DPWD mode embedded in this system has two independent sample volumes and can simultaneously record the flow velocity waves respectively. The two sample volumes (volume length 3.0mm) were located at the middle of the lumen along the observed artery. The direction and filter were adjusted according to the flow to get clear and satisfied flow spectra. The distance between two sites along the artery (ΔD, maximum as 45mm) could be directly measured on two-dimensional echography. The transit time (Δt) of the pulse wave between the two sites could also be obtained based on the spectra, where the time delay was measured by wave foot detection using the intersecting tangent method [22]. The PWV can be simply calculated as PWV = ΔD/Δt by this DPWD method.

### In vitro experiment

#### In vitro model

**Fig 1:**
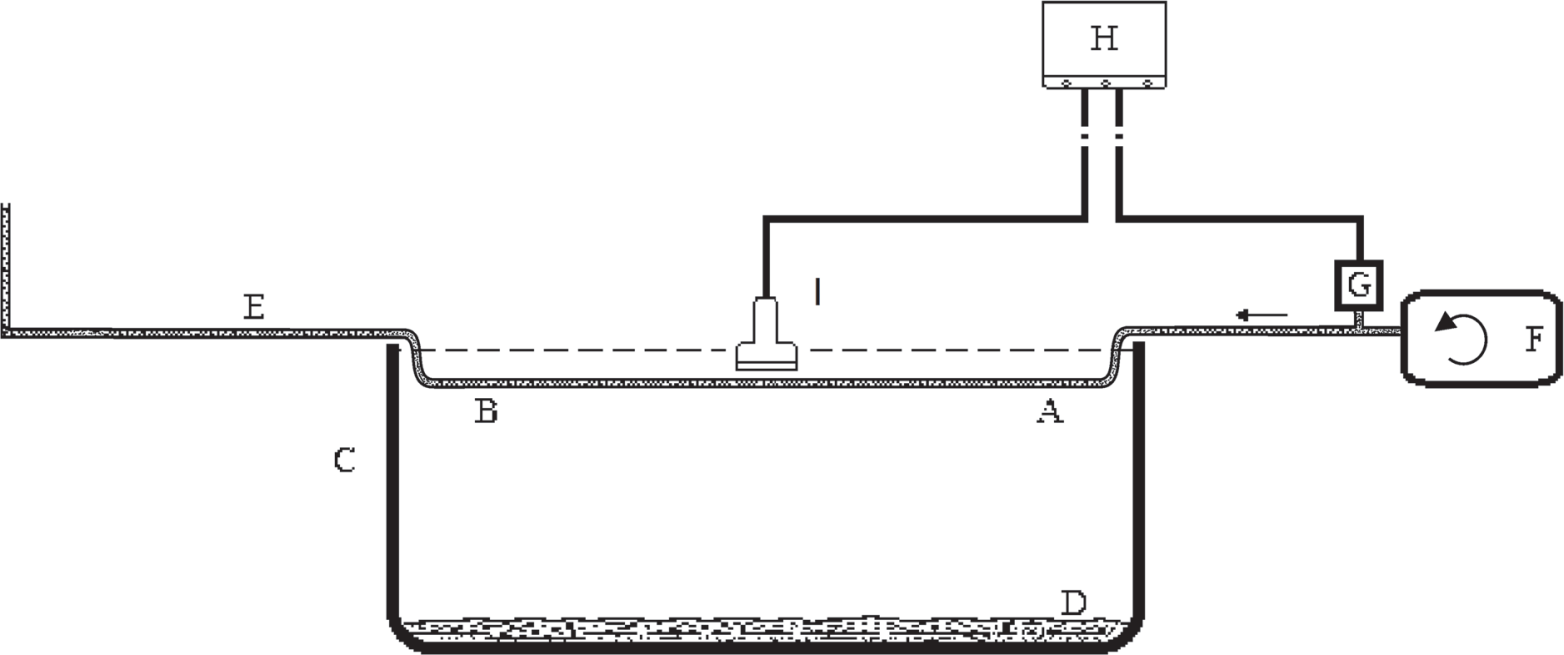
The sketch of the in vitro model. A and B, the two points chosen for the pulse wave velocity measurement; C, the water tank; D, a piece of sponge; E, the homogeneous rubber tubing; F, the perfusion pump; G, the pressure transducer; H, the ultrasound system; I, the linear array transducer. The pulse wave transit time between point A and B could be obtained by respectively recording the flow spectrum at point A and point B. The PWV of part A-B could be calculated as the distance between A and B could be easily measured. The local PWV of a short segment at the middle of point A and B could be measured using our method. And these two PWVs should theoretically be equal. PWV, pulse wave velocity.

To evaluate the precision and the accuracy of this new method, an in vitro experiment was designed (Fig. 1). This model consisted of a perfusion pump (YZ1515X Peristaltic Pump, Baoding Longer Precision Pump Co., Ltd, China) (F in Fig. 1)with variable pulse rate (0–5Hz/s) and a homogeneous rubber tubing E (internal diameter: 7mm), in which 5% (5g Fiber/100ml tap water) fiber-water slurry was driven through to simulate the blood flow within the artery. A pulse wave was generated by the perfusion pump and propagated along the homogeneous rubber tubing. The spectral flow velocity of the motion of the slurry could be recorded by pulse wave (PW) Doppler in the water tank. A pressure transducer G (MPS20N0020D_S, MEMStek Co., Ltd, Germany) was connected between the rubber tubing and the ultrasound system (H) near the beginning of the pulse wave by a T-branch pipe. The anode and cathode of the pressure transducer was respectively connected to the R-lead (right arm) and N-lead (left leg) of the embedded simultaneous ECG in this system. Thus, the pressure pulse curve could be simultaneously displayed at the position of routine ECG. The amplitude and filter of this pressure pulse curve could be adjusted in this system. The end of the rubber tubing was elevated in order to produce a base pressure in the system (20cm of height in this study). A piece of sponge (D) was placed at the bottom of the water tank (C) to absorb the extra ultrasound energy and reduce the interferences of the reflection.

#### Experimental principle and method

The ultrasound probe was positioned perpendicularly above the upstream point A of the rubber tubing. With the perfusion pumping, the slurry was driven and the pulse wave propagated along the rubber tubing. The flow at point A was recorded by PW Doppler. And the pressure curve was simultaneously recorded by the pressure transducer. This two spectra and pressure curve were simultaneously shown on the system. The transit time between the pressure transducer and point A (Δt_A_) was measured by identifying the onset of the pressure curve and the onset of the simulative flow spectrum (the foot) by intersecting tangent method. The transit time delay of the downstream point B (Δt_B_) was obtained with the same method. Thus, the difference of them was the transit time between point A and B. The distance between A and B (Δd_A-B_) could be measured directly. Therefore, the PWV of part A-B was calculated as PWV_A-B_ = Δd_A-B_ /(Δt_B_−Δt_A_).

The local PWV (PWV_M_) of a short segment (distance ranging from 39mm to 43mm, comparable with the in vivo study) at the middle of point A and B was measured using the DPWD method. Because of the homogeneity of the rubber tubing, It could be considered that local PWV_M_ equals to PWV_A-B._ Ten pairs of the PWV of the tubing with little different distance were measured and each measurement was averaged from 10 continuous measuring. The results of these two methods were compared in order to evaluate the reliability and accuracy of our new method.

### In vivo study

#### Subjects

Forty-five hypertensive patients (25male, 45 to 55years of age) were recruited from the Cardiology Section, Out-patient Department of Tangdu Hospital between January 2013 and June 2013. The age range was narrow in order to limit the factor of age. All the participants were selected by a general assessment based on the medical history inquiry, physical examination and routine echocardiographic examination. Inclusion criteria were: hypertension but free of other clinical cardiovascular diseases and any medication, heart rate from 60 to 80 bpm and nonsmokers. Exclusion criteria were: hyperlipidaemia, diabetes mellitus, arrhythmia, coronary heart disease or infection. And then 45 healthy subjects (25male, 45 to 55 years of age) were recruited from the staff of the hospital. They were similar in gender, weight, blood lipid, blood glucose and heart rate with the patients group. Hypertension was defined in patients as a systolic blood pressure ≧140 mm Hg and/or diastolic blood pressure ≧90 mm Hg. Hyperlipidaemia was defined as abnormally elevated fasting plasma cholesterol (total or low-density lipoprotein) levels. All the participants were asked to refrain from tea, alcohol and coffee for at least 24 h before the in vivo study. This study was approved by the Human Subjects Ethics Committee of Fourth Military Medical University. Written informed consent was obtained from each participant after the study was explained.

#### Study protocol

In this study, all participants adopted a supine position and had at least 10 min rest before any testing in a quiet, temperature-controlled (22±1°C) room in order to have a steady blood flow state and heart rate, according to the recommendations for standardization of methodological issues [11]. Before ultrasound examination, brachial blood pressure measurements were taken using an oscillometric device for 3 times, and the average was calculated. BP measurements were performed by a single investigator (Y. Y). The LCCA was selected for the local PWV measurement because it directly originated from the aortic arch and offered a straight and branch-free pathway until its bifurcation. The local section of interest of the artery and the two measurement sites along this artery were imaged by 2D echography with the artery kept horizontal at the maximum lumen, at a plaque-free area. Then we activated the PW/PW mode of the system, located the sample volume 1 at the middle of the proximal lumen about 5.5–6.0mm and sample volume 2 about 1.5–2.0mm upstream from the bifurcation with the same angle (< 60°) according to individual difference. Then the Doppler velocity spectra of blood flow at these two sites were simultaneously recorded. The transit time and the exact distance between two sites were measured. Ten heart beat measurements were taken and then averaged. The spectra were obtained and measured by one skillful sonographer (Z.W). During the measurements, the ultrasound transducer was applied gently to the skin surface with a minimum pressure, in order to eliminate the potential effects on blood flow and PWV. The ECG was recorded simultaneously in vivo.

Intra- and inter-observer variability of the measurements of local PWV were performed randomly in 20 hypertensive patients (10 male) and 20 healthy subjects (10 male) by two blinded observers (Z.W and Y.Y).

### Statistics

Continuous variables data were expressed as mean value±SD. The results of the PWV_A-B_ measured by the in vitro model and PWV_M_ by the new method were compared by paired *t* test after the normality test (using Shapiro-Wilk test). Other local PWV measurements and group profiles were compared with unpaired Student’s t test. Bland-Altman analysis and linear regression analysis were performed to determine precision and bias between the measurements. The coefficient of variation (CV) of the measurements in the in vitro and in vivo studies were calculated as the SD of the differences between paired measurements divided by the mean of all measurements. The results were analyzed using the statistical software SPSS 15.0 (SPSS, Chicago, IL, USA). A P value<0.05 was regarded as statistically significant.

## Results

### In vitro study

**Fig 2:**
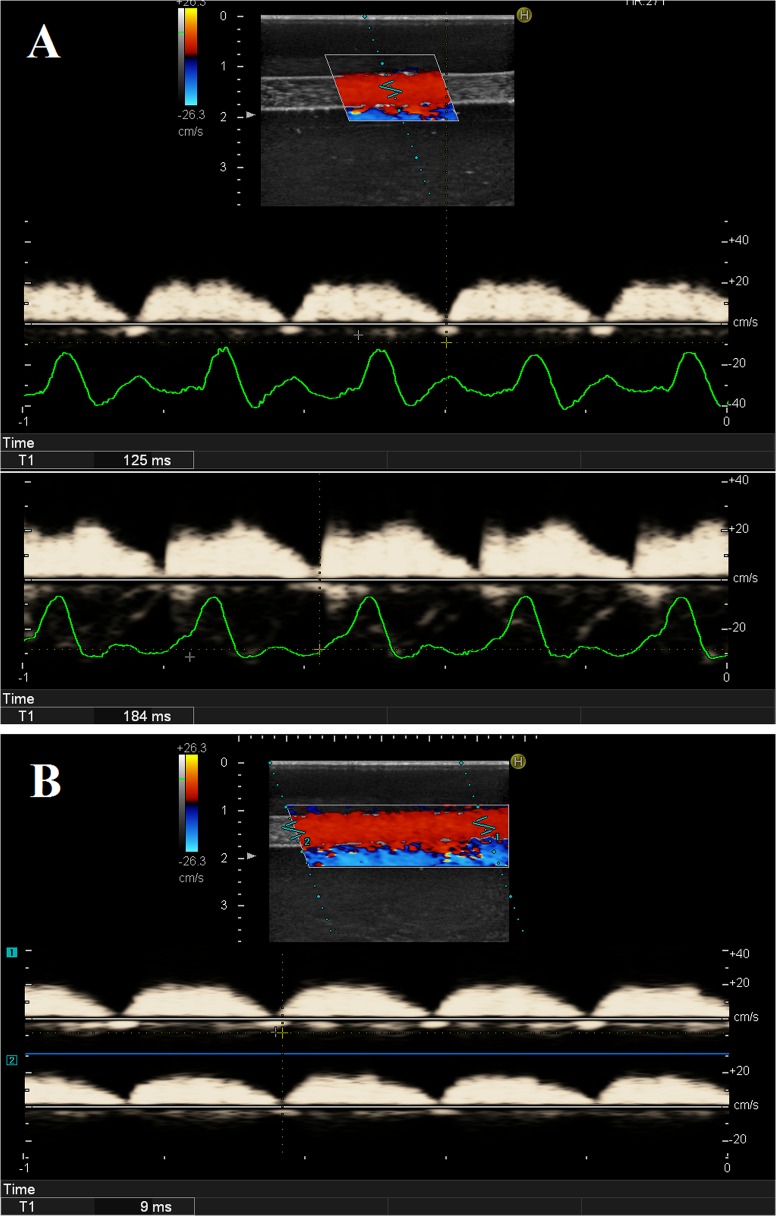
The methodology of the in vitro study. **Panel A,** the PWV_A-B_ measurement, the top panel was the two dimensional echocardiographic imaging. The velocity spectra recorded at point A and B by pulse wave Doppler were displayed at the middle and bottom, respectively. The pressure curve recorded by the pressure transducer was shown under each spectrum. The time delay between the transducer and point A and B were respectively measured by foot detection using the intersecting tangent method (125ms and 184ms). Thus the transit time between A and B could be calculated as 184−125 = 59ms. **Panel B,** the local PWV_M_ measured by the DPWD method, the simultaneously recorded dual velocity spectra were shown. The transit time between these two sites (9ms) was measured by wave foot detection using the intersecting tangent method. PWV_A-B_, pulse wave velocity between point A and B. DPWD, dual pulse wave Doppler; PWV_M_, pulse wave velocity at the middle of point A and B.

Ten pairs of PWV_A-B_ with different distance and PWV_M_ using the new method were measured. The in vitro experimental parameters and results of the PWV measurements are summarized in Table 1. Fig. 2 shows a representative PWV_A-B_ measurement and local PWV_M_ by the DPWD method based on the in vitro model. The mean value of the PWV between point A and B (PWV_A-B_ = 5.03 ± 0.15 m/s) and the local PWV of a short segment at middle (PWV_M_ = 5.16 ± 0.28 m/s) did not show difference with statistical significance (p = 0.075).

**Table 1:** The PWV measurements by the in vitro model.

	PWV_A-B_ Measurement			PWV_M_ Measurement		
Measurement	Δd_A-B_ (mm)	Δt_B_–Δt_A_ (ms)	PWV_A-B_ (m/s)	Δd_M_ (mm)	Δt_M_ (ms)	PWV_M_ (m/s)
1	389.3	74.3	5.24	38.4	7.1	5.41
2	380.1	75.2	5.05	38.4	7.4	5.19
3	376.4	73.9	5.09	39.7	8.0	4.96
4	366.0	76.4	4.79	41.2	8.6	4.79
5	351.0	73.0	4.81	40.4	8.6	4.70
6	349.6	69.6	5.02	39.6	7.3	5.42
7	320.7	64.9	4.94	41.2	8.1	5.09
8	313.1	61.9	5.06	38.4	6.9	5.57
9	301.0	58.7	5.13	39.8	7.7	5.17
10	287.4	55.6	5.17	40.7	7.7	5.29

Notes: PWV_A-B_, pulse wave velocity between point A and B; Δd_A-B_, the distance between A and B; Δt_A_, the transit time between the transducer and point A; Δt_B_, the transit time between the transducer and point B; PWV_M_, local pulse wave velocity at the middle of point A and B; Δd_M_, the distance of the chosen segment at the middle of point A and B; Δt_M_, transit time of the pulse wave at the middle segment.

**Fig 3:**
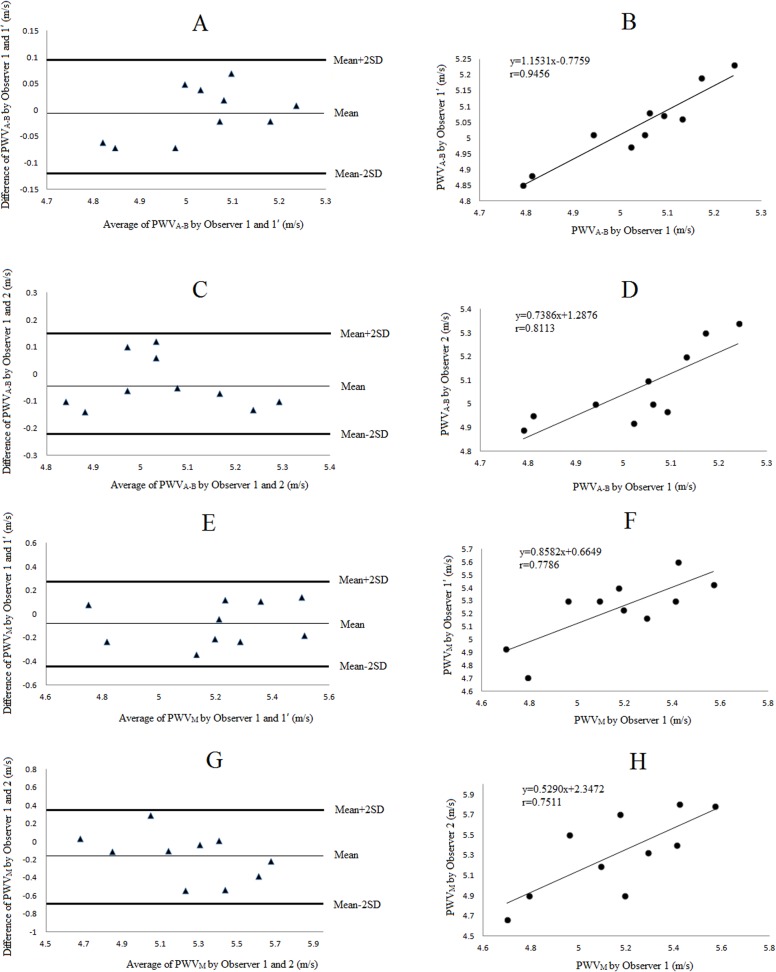
The reproducibilities of the PWV measurements in the in vitro experiment. Intra- (A, B) and inter-observer (C, D) variability of PWV_A-B_, intra- (E, F) and inter-observer (G, H) variability of PWV_M_ were demonstrated by Bland-Altman plots and linear regression analysis, respectively. Abbreviations as in Fig. 2.

We observed good agreement between measurements of PWV_A-B_ and PWV_M_ taken by the same and two independent observers. Fig. 3 and Table 2 showed the results of the reproducibility. The Bland-Altman plots and linear regression showed a good reproducibility, even though the PWV_M_ measurements had a relatively higher inter-observer variability (r = 0.75, CV = 4.96%).

**Table 2:** The reproducibility of the PWV measurements in vitro and in vivo studies.

In Vitro	Mean ± SD (m/s)	Mean Difference ± SD (m/s)	CV (%)
PWV_A-B_ (intra-observer)	5.03±0.13	−0.005±0.05	0.99
PWV_A-B_ (inter-observer)	5.05±0.15	−0.037±0.10	1.98
PWV_M_ (intra-observer)	5.20±0.25	−0.078±0.18	3.46
PWV_M_ (inter-observer)	5.24±0.32	−0.156±0.26	4.96
In Vivo			
PWV of healthy subjects (intra-observer)	5.31±0.72	0.001±0.11	2.07
PWV of healthy subjects (inter-observer)	5.31±0.74	−0.015±0.22	4.14
PWV of hypertensive patients (intra-observer)	6.30±1.01	0.013±0.14	2.22
PWV of hypertensive patients (inter-observer)	6.34±1.05	−0.081±0.25	3.94

Notes: PWV_A-B_, pulse wave velocity between point A and B; PWV_M_, local pulse wave velocity at the middle of point A and B; CV, coefficient of variation; SD, standard deviation.

### In vivo study

**Fig 4:**
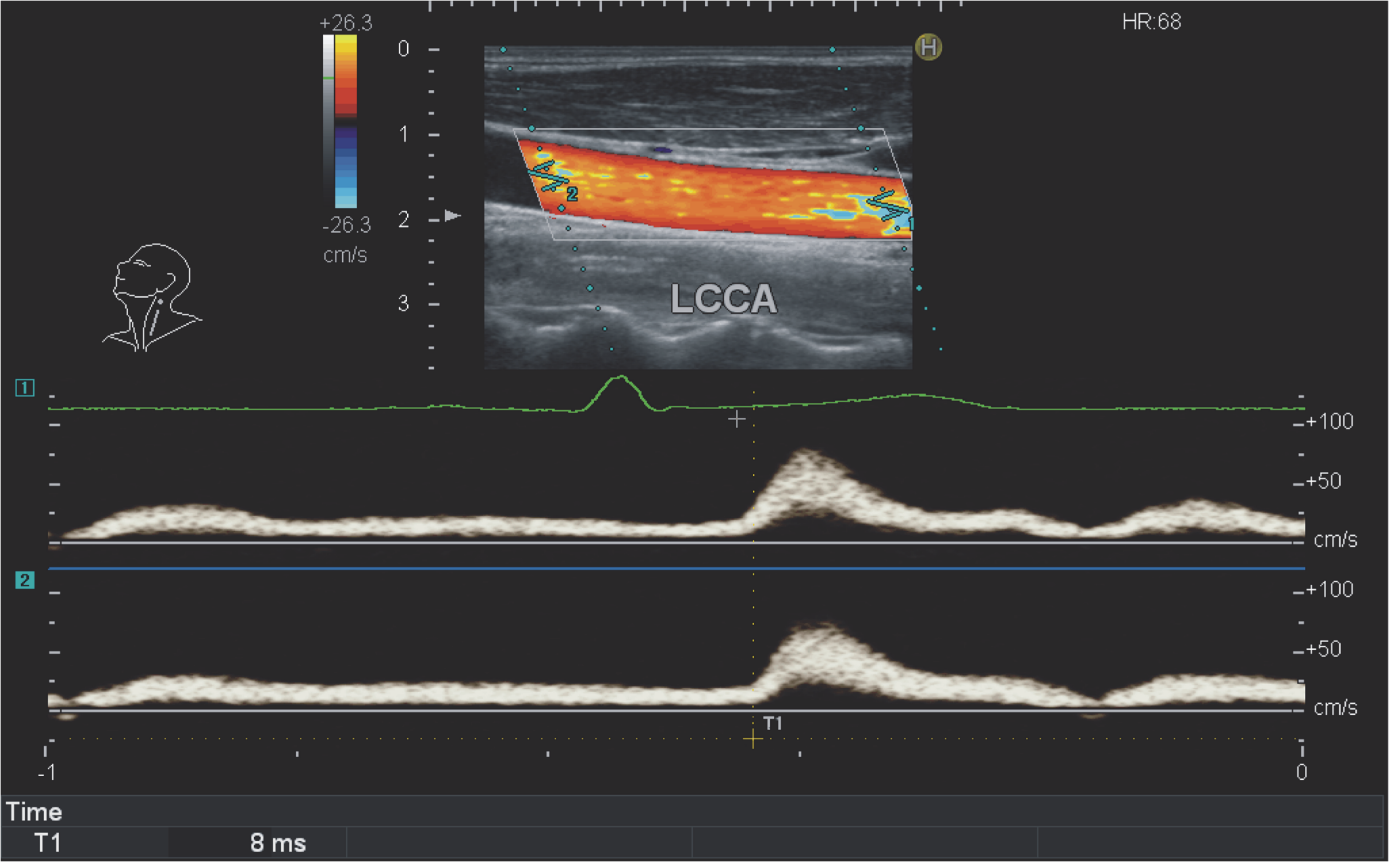
A representative local PWV measurement in a healthy subject. In the upper part, the two dimensional echography showed the upstream sample volume 1 and downstream sample volume 2 and their exact distance could be measured. The Doppler simultaneous velocity spectra were shown at the lower part. The transit time between these two sites was measured (8ms). LCCA, left common carotid artery; PWV, pulse wave velocity.

The sample characteristics are detailed in Table 3. Fig. 4 shows an example of local PWV measurement using DPWD method in a healthy subject. In hypertensive patients, the measured carotid artery local PWV values ranged from 5.09 m/s to 8.78 m/s and the mean value was 6.29±1.04m/s, which was higher than 5.31±0.72m/s (ranging from 4.23 m/s to 6.93 m/s) of the healthy subjects with a statistical significance (p = 0.019).

**Table 3:** The common characteristics of the participants in the in vivo study.

Parameters	Hypertension Patients (n = 45)	Healthy Subjects (n = 45)	P value
Age, yr	49.8±3.1	49.3±3.0	0.801
Sex, M/F	25/20	25/20	
Height, cm	169±10.2	170±10.4	0.230
Weight, kg	68.9±9.6	67.7±9.2	0.130
BMI, kg/m^2^	24.1±3.7	23.4±3.5	0.096
Brachial SBP, mm Hg	151±13	122±11	<0.001
Brachial DBP, mm Hg	96±8	76±6	<0.001
Brachial MBP, mm Hg	114±9	91±8	<0.001
HR, bpm	66±8	65±7.9	0.362

Notes: Values are means ± SD. BMI, Body Mass Index; SBP, systolic blood pressure; DBP, diastolic blood pressure; MBP, mean blood pressure; HR, heart rate.

**Fig 5:**
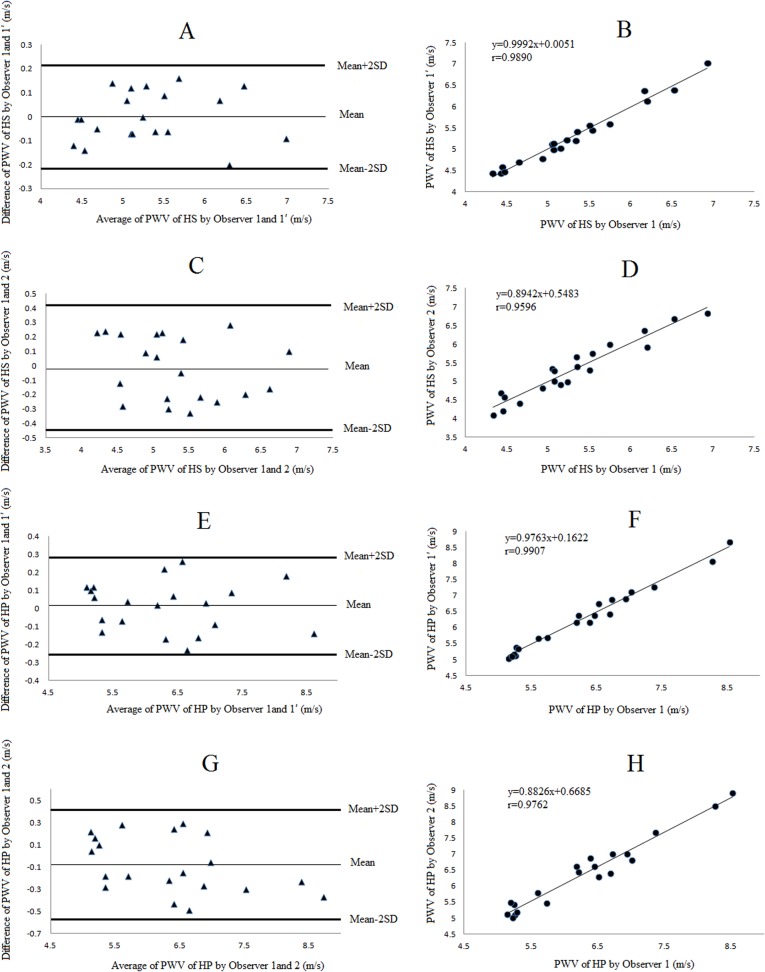
Intra- and inter-observer variability of the local PWV measurements in vivo. Intra- (A, B) and inter- (C, D) observer variability of local PWV measurements in healthy subjects (HS), intra- (E, F) and inter- (G, H) observer variability in hypertension patients (HP) were demonstrated by Bland-Altman plots and linear regression analysis, respectively.

The reproducibility of measurements of local PWV in healthy subjects and hypertensive patients taken by the same observer and two independent observers were shown in Table 2. The inter-observer variability of measurements in healthy subjects and hypertensive patients had relatively higher coefficient (4.14% and 3.94%), but still at a low level. The Bland-Altman plots and linear regression indicated a satisfactory reproducibility (Fig. 5).

## Discussion

In the current study, a new noninvasive, ultrasound-based DPWD method for local arterial PWV measurement was developed by in vitro and in vivo studies. The accuracy and reliability of the method was validated through the self-designed in vitro model. The in vivo study demonstrated that this clinical practical and reproducible method had the capability to detect an increased local PWV of LCCA in hypertensive patients from healthy subjects.

The aortic PWV, especially the carotid-femoral PWV, now as a reliable and reproducible prognostic measuring index or ‘gold-standard’ for arterial stiffness and used to predict cardiovascular events, has been extensively studied and well documented in various clinical conditions. However, two drawbacks of this method may hinder the further application in clinical practices. The first is its measuring inaccuracy caused by external surface distance measurement, which could not obtain the true length because of the anatomic particularities. The second is that this method could not evaluate biomechanical properties of a small segment or local artery wall. In clinical application, for example, in early stage atherosclerosis, small atherosclerotic plaques are scattered along the artery and the elastic properties of the locally involved artery wall will be affected [23]. In addition, Hickson SS and co-workers’ study [24] confirmed that aging had different impacts on stiffness of four regional aortic segments by regional PWV measurement using a magnetic resonance imaging technique. Therefore, measuring the local PWV has been of more interest in recent years, expecting to find diagnostic biomechanical information in some pathophysiologic conditions. All kinds of methods have been explored to fulfill this task, but these methods usually require specific devices and software and sometimes high technical expertise. Up to now, to our knowledge, there is no reliable method for local PWV measurement that could be directly used in clinical practice with accuracy and simplicity. The DPWD method we proposed in this study may be considered as a useful and convenient option for clinical local PWV measurement.

The in vivo study indicated that this new method was easy to perform without special devices in echo labs and could be grasped by clinical sonographer. The DPWD function of the ultrasound system has two independent sample volumes, which can be placed at the measurement sites according to 2D imaging guidance. The entire recording and measuring process could be done within 3 min. Another major advantage is that simultaneous ECG gating is not required for this new method, thus this method can be used in patients with arrhythmia or unstable heart rate subjects. The results of the in vivo study showed that the precision of the local PWV measurement was sufficient to distinguish hypertension group from healthy group. This new method could also be used in other arteries, such as ascending aorta, abdominal aorta, arteries of the limbs and so forth in different clinical settings to evaluate the cardiovascular risk and detect some incipient vascular diseases in the future studies.

The in vitro study may provide some useful information for the future related experiments. In the in vitro experiment, aiming to simultaneously record the simulative blood flow and pressure signal, we innovated to connect the pressure transducer with the ECG of the ultrasound system by the T-branch pipe. The pressure transducer converted pressure signal into voltage changing, with a maximum scale value of ±60 mV, which is comparable with the physiological ECG input level. The results of the in vitro study showed a good simultaneous flow velocity and pressure recording. In addition, in order to identify the foot of the pressure curve, the steep rise of the wave should be well displayed, we set the pulse rate of the perfusion pump at a high value of 4.5 Hz/s (nearly 4 times that of normal heart rate) based on series of preliminary experiments.

### Study Limitation

The commonly used method for measuring the transit time is the foot-to-foot method [11]. In order to measure the travel time of the foot of the wave over a short local artery, a high temporal resolution of the system is required, which is a common restriction for this temporal resolution related method. In this study, the dual PW mode of the ultrasound system has a relative low temporal resolution of 1ms. A small error in transit time measurement may cause a significant PWV deviation due to the short given distance. So, we averaged 10 consecutive measurements in this study. Of course, if the manufacturer develops an improved system with higher temporal resolution, for example, 0.1ms, the accuracy of the measurement related could be significantly improved. In this study, we did not divide the hypertension patients into different groups according to severity of the blood pressure, although most of the out-patients had mild hypertension. The local PWV of different levels of hypertension will be investigated in our further studies. In addition, the sample size of this study was small, but it was sufficient to achieve the aim to describe the methodology and evaluate its clinical applicability and reproducibility. In this study, existing methods have shown this method to be an important prognostic signal, and marker of local arterial stiffness, there is no doubt that new technique such as ours may be beneficial in the longer term. However, the work in this study is not sufficiently developed to make a compelling argument that the new method should be used to replace existing methods. Lots of further clinical studies are needed to demonstrate the value of this new approach.

## Conclusions

This study demonstrated the feasibility of the noninvasive DPWD method for determining the local PWV. The in vitro study indicates its accuracy and reproducibility. The in vivo study validates the clinical applicability and reproducibility and shows that the local PWV of common carotid artery is sufficiently precise to distinguish hypertensive patients from healthy subjects at the age of 44–55 years. This new echocardiographic method may be illuminating to clinical use and there should be a lot of work to be done.
